# Do acute effects of exercise on vascular function predict adaptation to training?

**DOI:** 10.1007/s00421-017-3724-8

**Published:** 2017-12-12

**Authors:** Ellen A. Dawson, N. Timothy Cable, Daniel J. Green, Dick H. J. Thijssen

**Affiliations:** 1 Research Institute for Sport and Exercise Science, Liverpool John Moores University, Tom Reilly Building, Byrom Street, Liverpool, L3 3AF UK; 2 Department of Physiology, Radboud University Nijmegen Medical Centre, Nijmegen, The Netherlands; 3 School of Sport Science, Exercise and Health, The University of Western Australia, Crawley, WA 6009 Australia; 4 Department of Sports Science, Aspire Academy, Doha, Qatar; 5 Principal Research Fellow, National Health and Medical Research Council of Australia, Canberra, Australia

**Keywords:** Exercise training, Vascular adaptation, Endothelial function

## Abstract

**Purpose:**

No previous study has explored the importance of exercise-induced changes in vascular function to prolonged adaptations. Therefore, the purpose was to explore the within-subject relationship between the acute post-exercise change in brachial artery endothelial function (flow-mediated dilation, FMD) and the change in resting FMD after a 2-week exercise training in healthy volunteers.

**Methods:**

Twenty one healthy, young men (24 ± 5 years) underwent assessment of brachial artery FMD using high-resolution ultrasound before and after 30-min of moderate-intensity cycle exercise (80% maximal heart rate). Subsequently, subjects performed five 30-min cycle exercise bouts at 80% maximal heart rate across a 2-week period, followed by repeat assessment of resting brachial FMD post-training.

**Results:**

Correcting for changes in diameter and shear, FMD did not change after the initial exercise bout (*P* = 0.26). However, a significant correlation was found between post-exercise changes in FMD and adaptation in resting FMD after training (*r* = 0.634, P = 0.002), where an acute decrease in post-exercise FMD resulted in a decrease in baseline FMD after 2 weeks and vice versa. We also found a positive correlation between antegrade shear rate during exercise and change in FMD% after acute exercise and after exercise training (*r* = 0.529 and 0.475, both *P* < 0.05).

**Conclusion:**

Our findings suggest that acute post-exercise changes in vascular function are related to changes in resting FMD after a 2-week endurance exercise training period in healthy men, an effect that may be related to exercise-induced increases in antegrade shear rate. This provides further insight into the relevance of acute changes in shear and FMD for subsequent adaptation.

## Introduction

Exercise training has strong cardioprotective effects in healthy asymptomatic individuals and in those with established cardiovascular disease (Booth et al. 2002). Although improvements in traditional (e.g. blood pressure, cholesterol) and novel cardiovascular risk factors (e.g. inflammatory markers) contribute to some of the cardioprotective effects of exercise training (Mora et al. 2007), a significant proportion (~ 40%) of the effect remains unexplained. Previous work suggests that direct effects of exercise on the arterial wall (Green et al. 2008; Joyner and Green 2009) can lead to adaptation in vascular function and structure that, at least partly, explain this “risk factor gap”. Studies have demonstrated that exercise training in subjects with cardiovascular risk or disease leads to improvements in function in coronary arteries (Hambrecht et al. 2000), conduit arteries (Watts et al. 2004; Thijssen et al. 2010), peripheral resistance arteries (Taddei et al. 2000; Maiorana et al. 2001) and skin microcirculation (Black et al. 2008b).

To understand the stimulus contributing to vascular adaptation following exercise training, studies have examined the acute impacts of single bouts of exercise on endothelial function. A review of this literature proposed that acute exercise leads to a biphasic response in endothelial function [measured as the flow-mediated dilation (FMD)] after intense exercise, with initial decreases in FMD superseded by return to normal or supra-normal levels 1–2 h after exercise (Dawson et al. 2013). This initial decrease may represent a hormesis stimulus, subsequently leading to improvement in resting vascular function. Indeed, previous work found that 2 weeks of exercise training is sufficient to induce improvement in resting FMD in healthy individuals (Tinken et al. 2008, 2009). However, to date, no previous study directly examined the relationship between acute vascular responses to exercise bout and subsequent training-induced vascular adaptation.

The purpose of our study was to relate acute change in brachial artery FMD after a single 30-min bout of cycle exercise to changes in resting brachial artery endothelial function following 2 weeks of cycle exercise training (involving five additional, similar exercise bouts). We hypothesized that an immediate decrease in FMD after the initial acute bout of exercise (pre- to post-first bout of exercise) is related to an increase in resting brachial artery FMD following 2 weeks of training (pre-exercise FMD before the first and final exercise bout). This work will provide more information on the potential relation between acute post-exercise change in vascular function and subsequent vascular adaptation.

## Methods

### Ethical approval and subjects

Twenty-two young male subjects (24 ± 5 years) were recruited from the University and local community for participation in this study. None of the subjects reported having been diagnosed with cardiovascular disease, diabetes mellitus type 1 or 2, insulin resistance or cardiovascular risk factors (such as hypercholesterolemia or hypertension). Subjects who smoked and/or were on medication of any type were excluded from participation. Informed written consent was obtained from all individual participants included in the study. The study procedures were approved by the Liverpool John Moores Ethics Committee and adhered to the Declaration of Helsinki. The study was performed during 2014–2015, with analysis and study write-up in 2016–2017.

### Experimental design

On day 1, brachial artery FMD was examined before and immediately after a 30-min cycling exercise bout performed at 80% of age-predicted maximal heart rate (Tanaka et al. 2001). At rest and during the final 10 min of exercise we assessed brachial artery diameter and velocity. From this we obtained information on resting and exercise-induced blood flow and shear rate patterns. Subsequently, subjects performed a 2-week exercise training programme (each session consisted of a 30-min exercise bout at 80% of maximal heart rate). After 2 weeks of exercise training (> 24 h after the final training session), we repeated the resting assessment of brachial artery FMD.

### Measurements

#### Baseline and post-exercise brachial artery endothelial function

On the first and final training session, subjects were instructed to abstain from strenuous exercise for 24 h and from caffeine and alcohol ingestion for 18 h before testing according to expert-consensus guidelines (Thijssen et al. 2011). Subjects were also instructed to fast for 6 h prior to each visit. Before baseline measurements, subjects rested in the supine position for 20 min in a temperature-controlled laboratory, followed by assessment of heart rate (HR) and blood pressure (BP) using an automated sphygmomanometer (GE Pro 300V2, Dinamap, Tampa, FL, USA). Assessment of brachial artery FMD was then undertaken. Immediately after the 30-min exercise bout, we repeated assessment of brachial artery FMD.

#### Exercise-induced blood flow and shear rate

Before and during the final 10 min of the first exercise bout, one arm was rested comfortably on an adjustable table. Duplex ultrasound (T3000; Terason, Burlington, MA, USA) was utilized to simultaneously image brachial artery diameter and velocity in one arm. We continuously measured diameter and red blood cell velocity during this time period.

#### Brachial artery flow-mediated dilation

This was assessed at least 24 h after the previous exercise bout. To examine brachial artery FMD, the arm was extended and positioned at an angle of ~ 80° from the torso. A rapid inflation and deflation pneumatic cuff (D.E. Hokanson, Bellevue, WA, USA) was positioned on the forearm, immediately distal to the olecranon process to provide a stimulus to forearm ischemia. A 12-MHz multi-frequency linear array probe, attached to a high-resolution ultrasound machine (T3000; Terason, Burlington, MA, USA) was then used to image the brachial artery in the distal 1/3rd of the upper arm. When an optimal image was obtained, the probe was held stable and the ultrasound parameters were set to optimize the longitudinal, B-mode image of the lumen–arterial wall interface. Continuous Doppler velocity assessments were also obtained using the ultrasound and were collected using the lowest possible insonation angle (always < 60°). Following baseline assessments, the forearm cuff were inflated (> 200 mmHg) for 5 min. Diameter and flow recordings resumed 30 s prior to cuff deflation and continued for 3 min thereafter, in accordance with technical specifications (Woodman et al. 2001; Black et al. 2008a).

#### Exercise training

Subjects cycled for five, 30-min exercise bouts over 2 weeks on an electrically braked ergometer (Monark 874E, Sweden), with resistance adjusted to maintain a stable and individually determined exercise intensity at 80% maximal heart rate (Tanaka et al. 2001). Subjects had a 2–5-min warm-up and then spent 30 min at 80% HR_max_. Heart rates were monitored throughout exercise using a Polar chest band and averaged across 5-min periods. Maximal heart rate was determined using a maximal incremental exercise test (*n* = 13) or adopting a widely accepted algorithm [*n* = 8, (Tanaka et al. 2001)]. For reasons unrelated to the current study, *n* = 11 underwent ischaemic preconditioning before each exercise bout. Nonetheless, for the purpose of this study, we ensured that subjects underwent 5 identical exercise bouts. Moreover, the purpose of the study was to determine if the within-subject acute response is related to the training stimulus.

### Data analysis

Analysis of brachial artery diameter was performed using custom-designed edge-detection and wall-tracking software, which is largely independent of investigator bias. Recent papers contain detailed descriptions of our analysis approach (Black et al. 2008a; Woodman et al. 2001). From synchronized diameter and velocity data, blood flow (the product of lumen cross- sectional area and Doppler velocity) was calculated at 30 Hz. Shear rate (SR, an estimate of shear stress without viscosity) was calculated as four times mean blood velocity/vessel diameter (Pyke and Tschakovsky 2005). The SR_auc_ (Shear Rate, area under the curve) was calculated for data from cuff release up to the point of maximal post-deflation diameter. Reproducibility of diameter measurements using this semi-automated software is significantly better than manual methods, reduces observer error significantly, and possesses an intra-observer CV of 6.7% (Woodman et al. 2001).

### Statistics

Changes from pre- to immediately post-exercise were determined using paired *t* tests. Given the possible change in baseline brachial artery diameter and post-deflation shear rate area-under-the-curve immediately following exercise (Dawson et al. 2013; Padilla et al. 2007), we also analysed the acute changes in FMD% using allometric scaling methods with a Generalized Estimating Equation, which included baseline artery diameter and shear rate area-under-the-curve as covariates (Birk et al. 2012; Atkinson et al. 2013). Changes from resting and exercise-induced blood flow and shear rate were assessed using paired *t* tests. The impact of exercise training on the outcome parameters was examined using paired *t* tests. To examine our primary aim, correlation between acute and chronic changes in FMD (both % and absolute diameter in mm) was assessed using Pearson’s correlation coefficient. We also correlated changes in FMD (both acute and training) with the exercise-induced increase in mean, antegrade and retrograde blood flow and shear rate and velocity. Statistical analysis was performed using SPSS 22.0 (SPSS, Chicago, Illinois). All data are reported as mean ± SD unless stated otherwise and level of significance was set at *P* < 0.05.

## Results

Twenty-two male subjects underwent all study procedures. Due to technical problems, we did not obtain a complete data set for one subject and he was subsequently removed from all analysis. The remaining 21 subjects were 24 ± 5 years, 79 ± 10 kg and 181 ± 9 cm.

### Exercise bout

We found that target HR (80% of maximal heart rate; 152 ± 4 bpm) was successfully achieved during exercise bouts, with average heart rate across all exercise sessions being 149 ± 6 bpm. Across the training sessions, we found no significant differences in the HR or workload between exercise sessions (Table 1). We found that mean, antegrade and retrograde shear and blood flow significantly increased during exercise in all subjects (Table 2).

**Table 1:** In-exercise heart rate (HR) and power during each training session

	Session 1	Session 2	Session 3	Session 4	Session 5	Total
HR (bpm)						
Total	149 ± 8	150 ± 8	149 ± 6	149 ± 9	148 ± 8	149 ± 6
Power (W)						
Total	149 ± 41	151 ± 43	150 ± 43	152 ± 43	153 ± 43	146 ± 52

Data are mean ± SD

Data for each session are the average from 5 to 30 min (data taken every 5 min)

**Table 2:** Brachial artery blood flow responses at rest and during final 10 min of first exercise bout

	Pre-exercise	During exercise
Mean BF (ml/min)	56 ± 37	253 ± 119^a^
Antegrade BF (ml/min)	70 ± 34	283 ± 115^a^
Retrograde BF (ml/min)	− 15 ± 11	− 30 ± 28^a^
Mean SR (s^−1^)	67 ± 33	283 ± 125^a^
Antegrade SR (s^−1^)	86 ± 29	316 ± 113^a^
Retrograde SR (s^−1^)	− 19 ± 13	− 33 ± 32^a^

Data are mean ± SD

*BF* blood flow, *SR* shear rate

^a^Significantly different from pre-exercise (*P* < 0.05)

### Post-exercise brachial artery flow-mediated dilation

There was a significant decrease in FMD immediately after the first bout of exercise from 5.1 ± 1.9 to 3.8 ± 1.9% (Table 3). We also observed a significant increase in baseline artery diameter and SR_auc_ during the immediately post-exercise FMD test (Table 3). There was no significant change in the time to peak dilation or peak dilation. When SR_auc_ and baseline artery diameter were included in the statistical model as co-factors, the immediate decrease in FMD after exercise was no longer statistically significant.

**Table 3:** Brachial artery characteristics before and immediately post-first exercise bout and after exercise training

	Pre-first exercise bout	Post-first exercise	Post-2-week exercise training
FMD (%)	5.1 ± 1.9	3.8 ± 1.9^a^	4.4 ± 1.9
Baseline diameter (mm)	4.1 ± 0.4	4.3 ± 0.4^a^	4.1 ± 0.5
Peak diameter (mm)	4.3 ± 0.4	4.4 ± 0.4	4.3 ± 0.5
TTP (s)	64 ± 24	77 ± 23	53 ± 17^a^
SR_auc_ (s^−1^ 10^3^)	18.6 ± 6.3	35.5 ± 15.1^a^	16.4 ± 3.8^a^

Data are mean ± SD

*FMD* flow-mediated dilatation, *TTP* time to peak dilation, *SR*_*auc*_ Shear rate area under the curve

^a^Significantly different from pre-first exercise bout

The acute change in FMD was significantly and positively correlated with exercise-induced changes in velocity (*P* = 0.05, *r* = 0.434), mean SR (*P* = 0.012, *r* = 0.538), antegrade SR (*P* = 0.014, *r* = 0.529), but not retrograde SR (*P* = 0.160, *r* = 0.318). Conversely, the acute change in FMD after a single exercise bout did not correlate with mean blood flow (*P* = 0.177, *r* = 0.307), antegrade blood flow (*P* = 0.235, *r* = 0.271) or retrograde blood flow (*P* = 0.215, *r* = 0.282).

### Relationship between acute and chronic changes

**Fig. 1:**
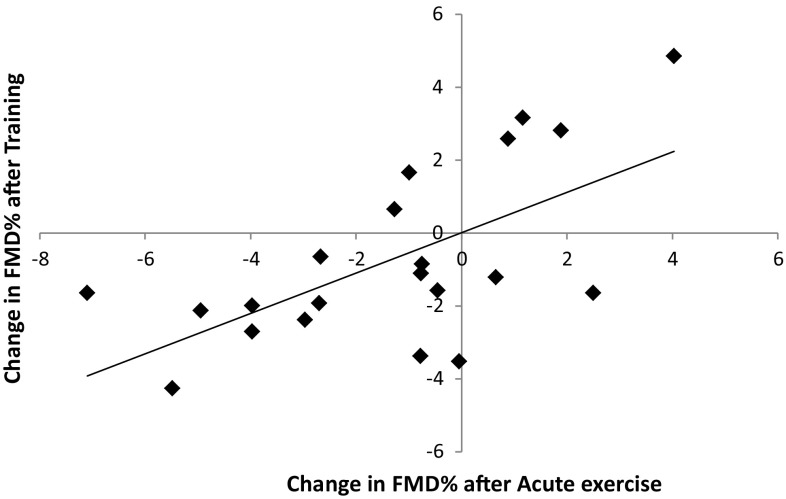
Correlation FMD% between post–pre and acute bout of exercise prior to training and post–pre-2 weeks of exercise training. *r* = 0.634, *P* = 0.002

Whilst there was as significant decrease in TTP and SR_auc_ after exercise training, there was no significant change in FMD after 2 weeks of exercise training (Table 3). There was, however, a significant positive correlation (*r* = 0.634, *P* = 0.002) between the immediate exercise-induced change (post–pre) in FMD versus the change in FMD after training (Fig. 1). This positive relation remained present when FMD was presented as the absolute diameter change in mm (*r* = 0.630, *P* = 0.002).

**Fig. 2:**
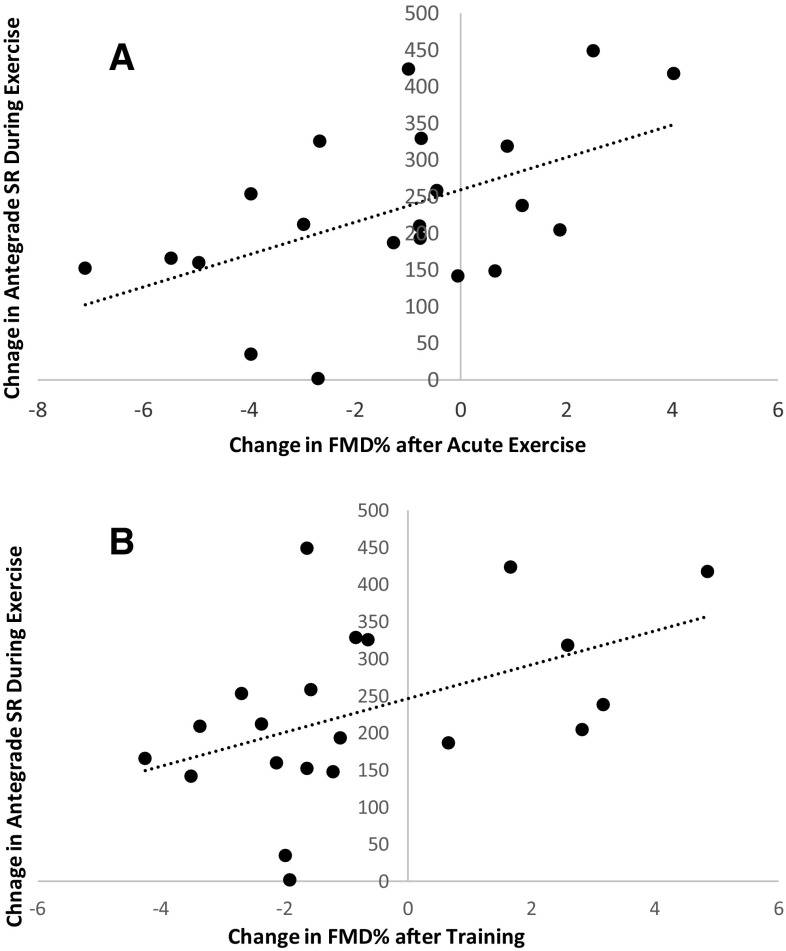
Correlation between FMD% post–pre and acute bout of exercise and antegrade SR during–pre the first exercise bout (upper panel, *r* = 0.526 *P* = 0.014) and FMD% post–pre-2 weeks of training and antegrade SR during–pre-first bout of exercise (lower panel, *r* = 0.475 *P* = 0.030)

The training-induced change in FMD was positively and significantly correlated to acute exercise-induced changes in mean SR (*P* = 0.021, *r* = 0.501), antegrade SR (*P* = 0.030, *r* = 0.475) (Fig. 2), but not retrograde SR (*P* = 0.098, *r* = 0.371). There was a trend for a correlation between velocity and training-induced FMD (*P* = 0.059, *r* = 0.419). There were no correlations between training-induced changes in FMD and exercise-induced changes in mean (*P* = 0.170, *r* = 0.311), antegrade (*P* = 0.247, *r* = 0.265) or retrograde blood flow (*P* = 0.143, *r* = 0.331). There were no changes in systolic (123 ± 9 mmHg versus 122 ± 8 mmHg) or diastolic blood pressure (66 ± 8 mmHg versus 65 ± 7 mmHg) after exercise training.

## Discussion

To our knowledge, this study provides the first data in humans to explore whether the changes in vascular function as a result of an acute bout of exercise are related to changes in vascular function following an exercise training programme. We found a strong positive relationship between acute post-exercise change in vascular function and the change in function following 2-week training. In contrast to the hormesis hypothesis, we noted a positive relationship between acute- and training-induced changes in vascular function which seem to be related to exercise-induced increases in shear rate. This would suggest that a ‘negative’ stimulus is not necessary to trigger a positive functional adaptation in the vasculature.

### Acute effect of exercise

After the first exercise bout, a decrease in FMD was observed. These findings are largely in line with previous work, in particular when examining the FMD immediately following an acute bout of moderate-to-strenuous intensity exercise of ≥ 30 min (Dawson et al. 2013). This decline is at least partly mediated through larger resting diameter post-exercise (Padilla et al. 2007), since statistically controlling for individual changes in baseline diameter removed the significant effect of an acute bout of exercise on FMD. The loss of significant decrease in corrected FMD% compared to previous studies may relate to exercise intensity and fitness of the subjects (Padilla et al. 2007; Dawson et al. 2013). Nonetheless, the minor increase in baseline artery diameter is unlikely to represent the only explanation for our observations (Gonzales et al. 2011; Birk et al. 2012) and exercise-induced increase in shear rate may also influence the post-exercise changes in FMD. This is supported by the significant positive correlation between increases in mean and antegrade shear rate during the exercise and pre–post-exercise changes in FMD, a finding in line with others (Tinken et al. 2009). Our findings provide further support for the notion that increases in mean or antegrade shear rate (Tinken et al. 2009; Whyte and Laughlin 2010) represent a potent stimulus for changes in vascular function. The change in shear rate during exercise is a result of a range of physiological factors including downstream resistance and central driving pressure. Consequently, changes in vasodilator mechanisms, blood pressure and cardiac output can all influence the shear stress response to exercise (Green et al. 2017). In addition, individual variations in sympathetic nervous activity and thermoregulatory response may help explain the between-subject variability in the exercise-induced changes in antegrade shear rate (Green et al. 2017; Benda et al. 2015).

It is pertinent to note that the change in FMD after acute exercise is both under the influence of positive (e.g. increasing shear rate) and negative (e.g. increasing oxidative stress and baseline diameter) perturbations (Whyte and Laughlin 2010; Padilla et al. 2007; Dawson et al. 2013). Therefore, the decrease in FMD in this study may be related to higher levels of exercise intensity and concomitant increase in oxidative stress (Johnson et al. 2012), activity of the sympathetic nervous system, increases in baseline diameter (Padilla et al. 2007) and/or marked elevation in blood pressure (Gonzales et al. 2011) which may mitigate the vasodilator effects of increases in shear. Taken together, our study provides further evidence for the presence of an immediate decrease in FMD after exercise when performed at moderate-to-high intensity.

### Acute effect of exercise versus adaptation to exercise training

We found a positive relation between acute changes in FMD versus adaptation in baseline FMD after 2 weeks of training, suggesting that an immediate decrease in function was related to a decrease in baseline function after training. The Hormesis hypothesis argues that low doses of an “agent” result in a biological improvement whereas high doses of this agent are toxic or damaging. The decrease in FMD following an acute bout of exercise may, therefore, unlikely be the “agent” underlying the adaptations that occur after 2-week exercise training. FMD is under a myriad of competing influences including shear stress, sympathetic activity, oxidative stress and artery size (Thijssen et al. 2011) and may, therefore, represent the outcome for a changing perturbation rather than representing a stimulus underling a prolonged adaptations per se. It is more likely that changes in stimuli during exercise may be the underlying agent, in particular shear stress during the exercise, a key stimulus which is strongly related to both acute and prolonged vascular adaptations (Laughlin et al. 2008; Whyte and Laughlin 2010). It is possible that high shear is damaging at high doses for those who are at risk or result in negative adaptations if normal patterns are disturbed (Eshtehardi et al. 2017; Davies 2009).

To further examine factors that may relate to the 2-week change in vascular function, we explored exercise-induced changes in blood flow and shear rate. Exercise-induced increases in shear stress are strongly related to an improvement in both vascular structure and function (Laughlin et al. 2008; Tinken et al. 2010). In support of this, changes in vascular function after exercise were significantly correlated to exercise-induced changes in mean and antegrade shear rate. More specifically, we found that large increases in shear rate during exercise were associated with large increases in FMD (Fig. 2). This relationship was apparent for both the acute changes in FMD after a single bout of exercise and the changes in FMD after 2 weeks of training. The correlations found with shear and velocity suggest that this correlation is not driven by a change in baseline diameter which is used in the calculation of both shear and FMD. This observation provides further support for an important role for exercise-induced elevations in shear rate in mediating vascular adaptation. Therefore, exercise-induced changes in shear rate may underlie the strong, positive relation between acute and chronic changes in vascular function. Further work is needed to determine if similar relationships are found with longer training programme, more intense exercise, in subjects with different activity/fitness levels or if it is necessary for structural rather than functional adaptations.

### Limitations

There are a number of methodological issues that may have influenced our findings. Since we only assessed cycling exercise in healthy subjects, we cannot extrapolate our findings to different types or modes of exercise or to different populations. Similarly, we did not include a measurement of fitness, as training has been shown to influence the acute and chronic vascular responses (Dawson et al. 2013; Green et al. 2011). Likewise, additional stimuli to shear stress could be mediating the response including changes in oxidative stress (Padilla et al. 2011) or alterations in other hemostatic and inflammatory variables, including blood pressure which was not assessed post-exercise (Whyte and Laughlin 2010). The training duration was relatively short and whilst we have previously found improvements in healthy individuals after 2 weeks (Tinken et al. 2010), that was not apparent in this study. This relationship between acute changes in FMD and subsequent adaptation may, therefore, change with longer duration training. We did not have a control group in this study, although all comparisons are made within subjects. Time of day may influence FMD (Jones et al. 2009; Al Mheid et al. 2014), although findings are equivocal (Kim et al. 2015). Consequently, all efforts were made to retest all subjects at the same time of the day for all repeat measurements. However, we were not able to guarantee this in all subjects due to difficulties in availability and testing schedules. Nonetheless, our novel observations of a potential link between acute and chronic changes in vascular function warrant future studies to explore this possible relation.

In conclusion, this is the first study to explore and identify a relationship between acute and chronic changes in vascular function to exercise and training. We report a positive correlation between changes in FMD following an initial bout of cycle endurance exercise and the adaptation in baseline FMD after 2 weeks of training. Possibly, this relationship may be underpinned by individual changes in shear rate during each exercise bout. More specifically, a large increase in shear during exercise was related with an increase in post-exercise FMD, whilst this latter response was associated with improved baseline FMD after 2 weeks of exercise training. Therefore, these data provide further support for the key role of (repeated) increases in antegrade and mean shear rate as a stimulus for adaptation in vascular function. The acute responses to exercise may be helpful in understanding (and predicting) future vascular adaptation in function and structure when repeating the exercise.

All procedures performed in studies involving human participants were in accordance with the ethical standards of the institutional and/or national research committee and with the 1964 Helsinki declaration and its later amendments or comparable ethical standards.
